# Evaluation of a clinical score to predict post-sclerotherapy hyperpigmentation compared with the Skin Hyperpigmentation Index: a prospective Skin Code study

**DOI:** 10.1590/1677-5449.202501082

**Published:** 2026-09-28

**Authors:** Renata Camila Barros Rodrigues, Samantha Neves, Eduardo Ramacciotti, Viviane Santana da Silva, Roberto Augusto Caffaro, Flávia Magalhães Silveira Magella Oliveira, Alexandre Fioranelli, Rebeca Higino Becker

**Affiliations:** 1 Faculdade de Ciências Médicas da Santa Casa de Misericordia de São Paulo, São Paulo, SP, Brasil.; 2 Science Valley Research Institute, São Paulo, SP, Brasil.; 3 Hospital Alemão Oswaldo Cruz, São Paulo, SP, Brasil.; 4 Instituto de Infectologia Emílio Ribas, São Paulo, SP, Brasil.; 5 Ambulatório Médico de Especialidades Dr. Geraldo Paulo Bourroul, São Paulo, SP, Brasil.

**Keywords:** post-inflammatory hyperpigmentation, telangiectasias, reticular veins, sclerotherapy, skin, hiperpigmentação pós-escleroterapia, telangiectasias, veias reticulares, escleroterapia, pele

## Abstract

Post-sclerotherapy hyperpigmentation (PSH) is a common complication following sclerotherapy for telangiectasias and reticular veins. However, tools for pre-procedural prediction of PSH are limited, making pre-treatment identification of patients at greater risk a challenge. This research aimed to evaluate a clinical scoring system (Skin Code Score) designed to estimate the individual risk of PSH. A prospective, single-center, single-arm study was conducted. Adult female patients with telangiectasias and reticular veins were treated and evaluated for pigmentation risk using the Skin Code Score (range, 2-12 points). Hyperpigmentation was assessed at baseline and on day 30 by in-person evaluation performed by one vascular surgeon, complemented by photographic analysis conducted independently by two other vascular surgeons and one dermatologist. Skin Hyperpigmentation Index (SHI) measurements were used for objective correlation with the Skin Code Score. The Skin Code Score showed strong interobserver agreement and reproducibility (κ ≥ 0.85). Diagnostic accuracy ranged from 82.8% to 94.7% among evaluators. A score of 12 predicted PSH in up to 99.8% of cases, while a score of 8 indicated a probability of approximately 76%. The study was limited by a small sample size and its single-center design; a proportion of the assessments were conducted by photographic analysis. It is concluded that the Skin Code Score is a simple and reproducible tool for predicting PSH and could assist clinicians with pre-procedural risk stratification, patient counseling, and clinical decision-making.

## Resumo

A hiperpigmentação pós-escleroterapia (HPE) é uma complicação comum após a escleroterapia de telangiectasias e veias reticulares. No entanto, os instrumentos para predição pré-procedimento da HPE são limitados, tornando desafiadora a identificação de pacientes com maior risco antes do tratamento. A pesquisa objetivou avaliar um sistema de escore clínico (Skin Code Score) desenvolvido para estimar o risco individual de HPE. Foi realizado um estudo prospectivo, unicêntrico e de braço único. Pacientes adultas do sexo feminino com telangiectasias e veias reticulares foram tratadas e avaliadas quanto ao risco de pigmentação utilizando o Skin Code Score (intervalo de 2 a 12 pontos). A hiperpigmentação foi avaliada no 1° dia e no 30º dia por meio de exame presencial realizado por um cirurgião vascular, complementado por análise fotográfica conduzida de forma independente por dois cirurgiões vasculares e um dermatologista. Medidas do Índice de Hiperpigmentação Cutânea (Skin Hyperpigmentation Index – SHI) foram utilizadas para correlação objetiva com o Skin Code Score. O Skin Code Score apresentou forte concordância interobservador e reprodutibilidade (κ ≥ 0,85). A acurácia diagnóstica variou de 82,8% a 94,7% entre os avaliadores. Um escore de 12 previu HPE em até 99,8% dos casos, enquanto um escore de 8 indicou probabilidade aproximada de 76%. O estudo foi limitado pelo pequeno tamanho amostral e pelo delineamento unicêntrico; parte das avaliações foi realizada por meio de análise fotográfica. Conclui-se que o Skin Code Score é uma ferramenta simples e reprodutível para a predição de HPE e pode auxiliar os médicos na estratificação de risco pré-procedimento, no aconselhamento dos pacientes e na tomada de decisão clínica.

## INTRODUCTION

Post-sclerotherapy hyperpigmentation (PSH) is a specific subtype of post-inflammatory hyperpigmentation and constitutes the most common adverse effect following sclerotherapy for telangiectasias and reticular veins of the lower limbs. Despite being considered a benign complication, PSH may compromise aesthetic outcomes, negatively impact patient satisfaction, and, in some cases, persist for prolonged periods, requiring additional therapeutic interventions.^1-7^

The pathophysiology of PSH involves endothelial injury induced by sclerosing agents, leading to inflammation, erythrocyte extravasation, hemosiderin deposition, and stimulation of melanocytes, resulting in increased cutaneous melanin production. Previous studies report PSH rates ranging from 10% to 30%, with approximately 10% of cases persisting beyond one year. This variability suggests that individual susceptibility plays a relevant role in the development of pigmentation following sclerotherapy.^8-11^

Several patient-related factors have been associated with an increased risk of PSH, including higher skin phototypes, pre-existing pigmentation in the treated area, recent tanning, and darker palmar crease coloration. Tools such as the Fitzpatrick scale and palmar crease pigmentation assessment have been proposed to assist clinicians in estimating pigmentation risk. However, these approaches remain qualitative and subject to interobserver variability, limiting their predictive accuracy in routine clinical practice.^11-21^

In recent years, objective methods for quantifying skin pigmentation have been developed to improve the assessment of hyperpigmentation after its occurrence. The Skin Hyperpigmentation Index (SHI) is an automated tool that quantifies skin hyperpigmentation through digital image analysis, comparing the affected area with the patient’s normal skin. The index ranges from 1 (no pigmentation) to 4 (intense pigmentation) and uses digital image processing to ensure an objective and reliable evaluation.^12^ The system is simple and accessible: healthcare professionals capture skin images using a smartphone or a standardized camera and upload them to the online platform, where the index is automatically generated within seconds. Among its advantages, the SHI ensures objectivity, standardization, and speed, making hyperpigmentation monitoring more dependable and improving clinical follow-up and patient communication.^10,22,23^

Although the SHI constitutes an important advance in standardization of post-treatment evaluation, it is primarily designed to measure hyperpigmentation after it has developed and does not offer a pre-procedural risk stratification tool.^22,23^

Therefore, despite the existence of objective methods to assess hyperpigmentation outcomes, there remains a lack of simple, clinically applicable instruments capable of predicting individual risk of PSH before sclerotherapy. This gap limits personalized patient counseling, informed consent, and the implementation of preventive strategies tailored to patients at higher risk.^23^

## METHODS

### Study design and oversight

This investigator-initiated, non-randomized*,* prospective, single-center, single-arm study was designed to evaluate the Skin Code Score as a predictive tool for PSH in women with telangiectasias and reticular veins. Following standardized clinical and photographic assessments, the Skin Code study was conducted in Ji-Paraná, Brazil, between March and December 2023. The first and senior authors designed and led the study, with all co-authors contributing to protocol refinement and manuscript preparation. No external writing assistance was provided.

The National Research Ethics Committee (CONEP) of the Brazilian Ministry of Health approved the study protocol, with CAAE submission certificate 65651422.6.0000.9147 and Ethical Approval Code number 5.920.068. The study complies with the protocol and fully adheres to the ethical principles of the Declaration of Helsinki and the International Council for Harmonization, and Good Clinical Practice guidelines. The protocol requires each patient to provide informed consent before beginning any study procedures.

### Inclusion and exclusion criteria

Eligible participants were women aged 18-69 with chronic venous disease (CVD) C1 CEAP (clinical, etiological, anatomical, and pathophysiological) classification (with telangiectasias and reticular veins), who were willing to sign the informed consent.

Exclusion criteria included individuals with CVD CEAP 2-6 classifications, arterial disease, history of deep vein thrombosis, comorbidities such as diabetes mellitus, heart failure, systemic arterial hypertension, thyroid disorders, allergy to polidocanol, dermatitis (or other lower limb skin condition), hypercoagulable states, asthma, migraines, use of anticoagulants, pregnant or lactating women, and those who did not provide consent.

### Study procedures

Patients attended a private outpatient clinic specialized in angiology and vascular surgery with elective care and provided written informed consent before enrolment. Baseline clinical data were collected, including demographics, medical history, and skin characteristics. The 21-patient sample was selected by convenience and comprised consecutive, non-randomized patients.

On day 1, to ensure image consistency, all patients were photographed using an iPhone^®^14 under semi-standardized artificial lighting. The lead author classified patients using the Skin Code Score, ranging from 2 to 12 points. Sclerotherapy was performed using liquid polidocanol (0.25% for telangiectasias and 0.5% for reticular veins), with a maximum of 0.3 mL per injection and 10 mL per session until the veins cleared.

On day 30, the lead author reassessed the patients in person. At the same time, three additional evaluators (one dermatologist and two vascular surgeons) independently analyzed 42 standardized photographs forwarded by Google Forms^®^(pre- and post-treatment images of 21 patients) (Figure 1).

**Figure 1:**
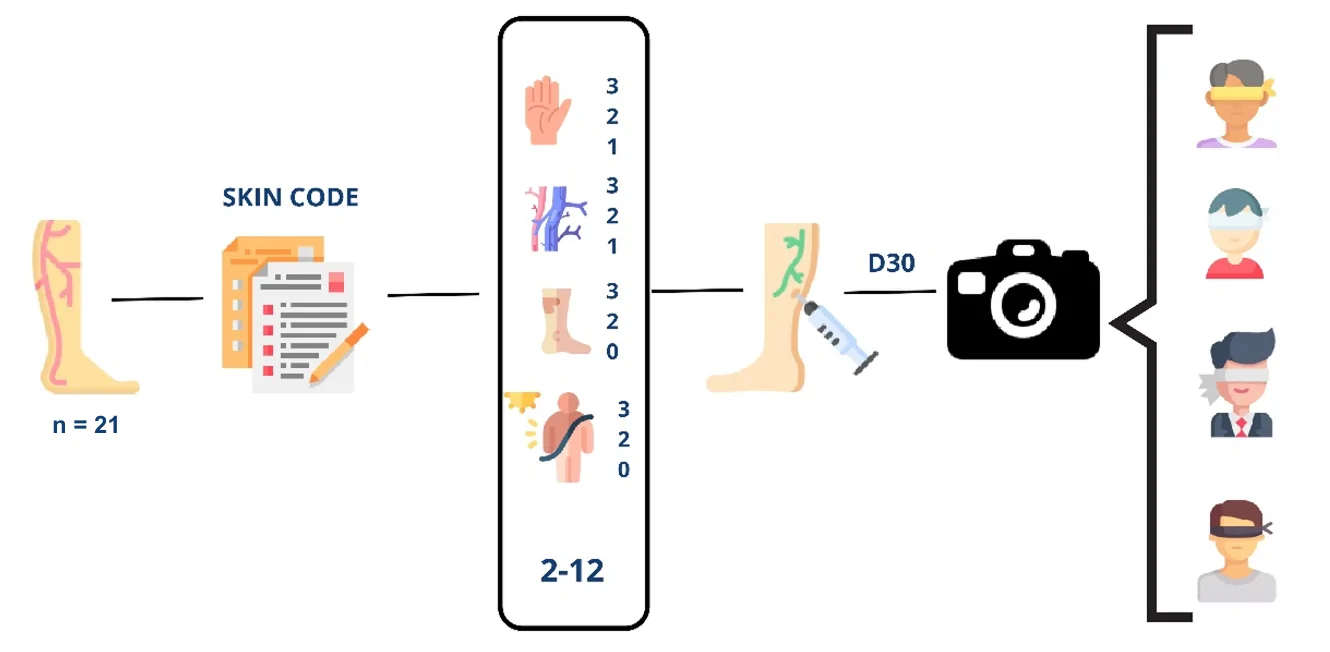
Study design.

Hyperpigmentation was assessed at baseline and 30 days post-treatment by four independent physicians (one dermatologist and three vascular surgeons) using the Skin Code Score.

### Skin Code Score

The Skin Code Score is a clinical scoring system developed to estimate an individual’s risk of developing hyperpigmentation after sclerotherapy. The score combines four clinically assessable variables, each supported by prior literature: skin tanning status, type of venous lesion treated (telangiectasias or reticular veins), presence of pre-existing pigmentation in the treatment area, and palmar crease pigmentation.

Palmar crease color was evaluated according to the palmar crease pigmentation scale described by Leal-Silva,^21^ classifying pigmentation as either red or brown. Venous lesion type was determined by clinical examination following CEAP definitions, distinguishing telangiectasias from reticular veins based on their depth and differing potential for hemoglobin extravasation.

Each variable was assigned a predefined score, yielding a total Skin Code Score ranging from 2 to 12 points. Higher scores indicate a greater predicted risk of post-sclerotherapy hyperpigmentation. The full scoring system is presented in Chart 1.

**Chart 1:** *Skin Code Score*.

**PALMAR CREASE COLOR**	
Brown palmar crease	3
Palmar crease intermediate color	2
Red palmar crease	1
**VESSEL TO BE TREATED**	
Reticular veins and telangiectasias	3
Reticular veins	2
Telangiectasias	1
**STAIN**	
Brown pre-stain	3
Intermediate color pre-stain	2
No prior stain	0
**SKIN TANNING**	
Tanned skin	3
Inconclusive whether skin tanned	2
Skin not tanned	0
**TOTAL**	**2-12**

### Skin Hyperpigmentation Index (SHI)

The SHI is an automated tool that quantifies skin hyperpigmentation through digital image analysis, comparing the affected area with the patient’s normal skin. The index ranges from 1 (no pigmentation) to 4 (intense pigmentation) and uses digital image processing to ensure an objective and reliable evaluation.^12^ The system is simple and accessible: healthcare professionals capture skin images using a smartphone or a standardized camera and upload them to the online platform, where the index is automatically generated within seconds. Among its advantages, the SHI ensures objectivity, standardization, and speed, making hyperpigmentation monitoring more dependable and improving clinical follow-up and patient communication.^10,22,23^

## OBJECTIVES

### Primary objective

The main objective of the study was to evaluate the clinical applicability of a scoring system, the Skin Code Score, to determine the likelihood of PSH of reticular veins and telangiectasias of the lower limbs.

### Secondary objectives

To evaluate the effectiveness of the Skin Code Score for predicting PSH in patients with CVD CEAP 1 undergoing sclerotherapy.

To evaluate the agreement between the scores obtained by the Skin Code Score and the SHI calculator for predicting PSH in patients with CVD CEAP 1 undergoing sclerotherapy.

### Statistical analysis

Interobserver agreement was assessed using the Kappa Fleiss coefficient (κ). Diagnostic accuracy was calculated by comparing the evaluators’ classifications with those of the gold standard specialist. Statistical significance was set at p < 0.05. The relationship between the Skin Code Score and the SHI was analyzed using Pearson correlation coefficients. All analyses and graphical outputs were produced using RStudio^®^ (RStudio, PBC, Boston, 2020).

## RESULTS

### Patient characteristics and study design

The Skin Code study was a prospective, single-center, single-arm study conducted between March 2023 and December 2023 to evaluate the relationship between the Skin Code Score and post-sclerotherapy hyperpigmentation, and its reproducibility. Initially, 135 individuals were screened, 114 of whom were excluded because they did not meet the inclusion criteria (n = 104) or refused to take part (n = 10). As a result, 21 white female patients with telangiectasias and reticular veins in the lower limbs were recruited, underwent treatment, and completed the follow-up with no losses or dropouts (Figure 2). Mean age was 27.6 years (standard deviation of 9.1 years) and other demographic data are shown in Table 1.

**Figure 2:**
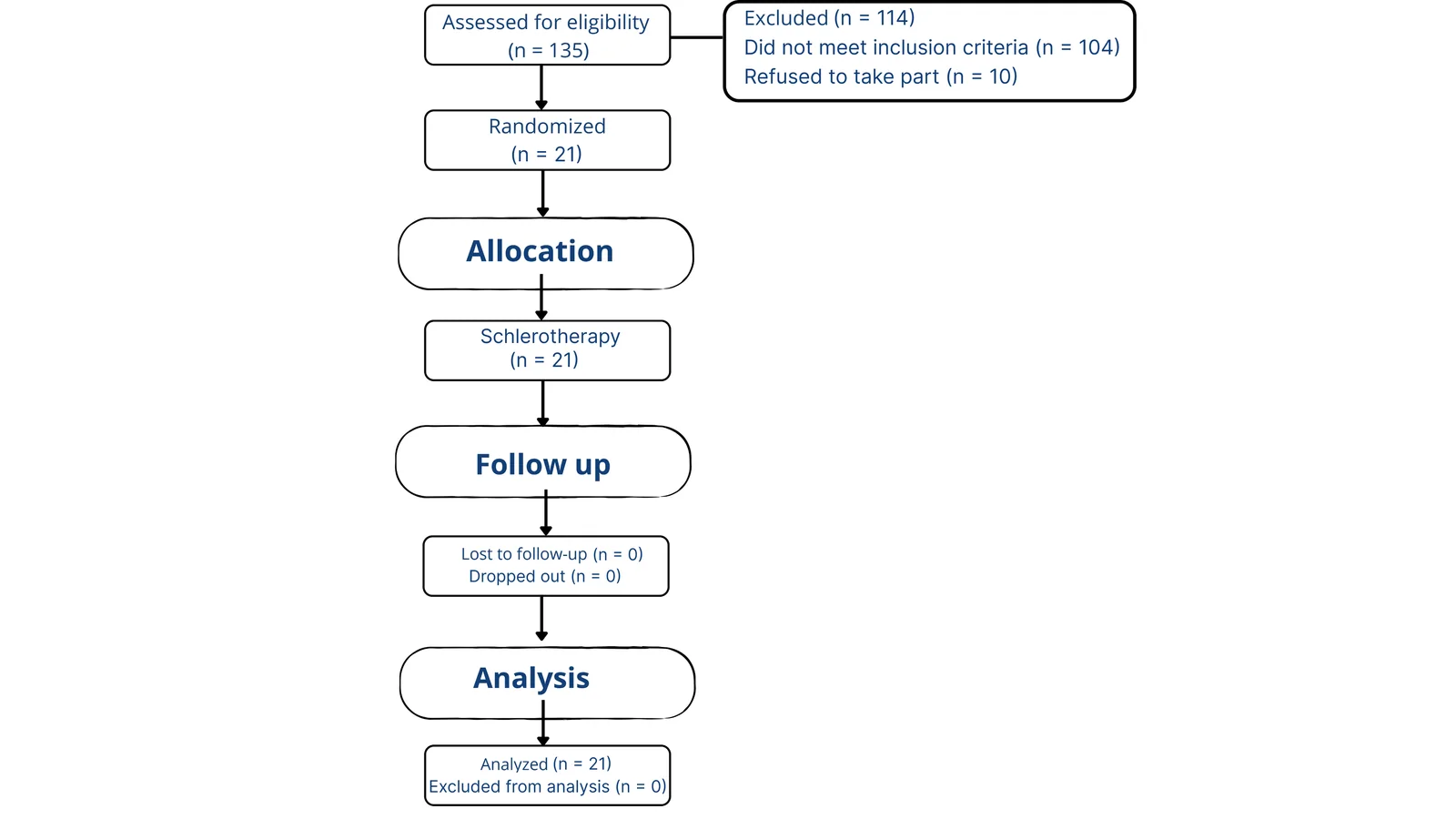
Consort flowchart.

**Table 1:** Demographic and clinical characteristics.

**Variable**	**Category**	**n (%)**
Gender	Female	21 (100%)
Age (years)	20-30	4 (19.0%)
	31-40	13 (61.9%)
	41-50	2 (9.5%)
	51-60	2 (9.5%)
Body Mass Index (BMI)	18.5-24.9	17 (80.9%)
	25-29.9	4 (19.1%)
Physical activity	Sedentary lifestyle	11 (52.3%)
	Once a week	1 (4.9%)
	Three times a week	9 (42.8%)
Obstetric history	Nulliparous	8 (38.2%)
	G1	4 (19.0%)
	G2 or more	9 (42.8%)
Personal history	Dyslipidemia	1 (4.9%)
	Anxiety	2 (9.5%)
Palmar crease color	Brown	13 (61.9%)
	Intermediate	6 (28.6%)
	Red	2 (9.5%)

Legend: Categorical variables are presented as absolute numbers (*n*) and percentages (%). Source: Skin Code Score Study.

### Interobserver agreement and diagnostic sensitivity

The κ coefficient demonstrated strong interobserver agreement among evaluators, with values ranging from 0.85 (BD) to 0.91 (AC and AD), all statistically significant (p<0.05). The diagnostic accuracy of the Skin Code Score remained consistently high, ranging from 82.8% (AB) to 94.67% (AC), with minimal variation between evaluators (1.05% to 2.02%). Following the pre-evaluation calibration test, evaluators’ responses showed over 99% reliability and bias below 1% across all assessments (p < 0.05). These findings demonstrate that the agreement metrics are robust and not attributable to chance, as evidenced by the statistically significant p-values presented in Table 2 and Table 3 and Figure 3.

**Table 2:** Interobserver Agreement (Kappa, p-value).

**Interobserver Agreement**	**Visual examination – hyperpigmentation (yes/no)**
A × B	*κ = 0.89 (p < 0.001)*
A × C	*κ = 0.90 (p < 0.001)*
A × D	*κ = 0.91 (p < 0.001)*
B × C	*κ = 0.82 (p < 0.001)*
B × D	*κ = 0.85 (p < 0.001)*
C × D	*κ = 0.88 (p < 0.001)*

Legend: Interobserver agreement for pigmentation assessment. (A) dermatologist; (B) principal investigator; (C) vascular surgeon; (D) vascular surgeon. Source: Skin Code Score Study.

**Table 3:** Observed hyperpigmentation classification (n, %).

**Observer**	**Present n (%)**	**Absent n (%)**
**Visual Evaluation**		
A (dermatologist, gold standard)	13 (62.0)	8 (38.0)
B (PI)	4 (19.0)	17 (81.0)
C (vascular surgeon)	11 (52.4)	10 (47.6)
D (vascular surgeon)	11 (52.4)	10 (47.6)
**Objective Evaluation – SHI (PI)**		
B (PI)	6 (28.6)	15 (71.4)

Legend: Data are presented as absolute numbers (n) and percentages (%). (A) dermatologist (gold standard); (B) principal investigator (PI); (C) and (D) vascular surgeons. SHI: Skin Hyperpigmentation Index. Source: Skin Code Score Study.

**Figure 3:**
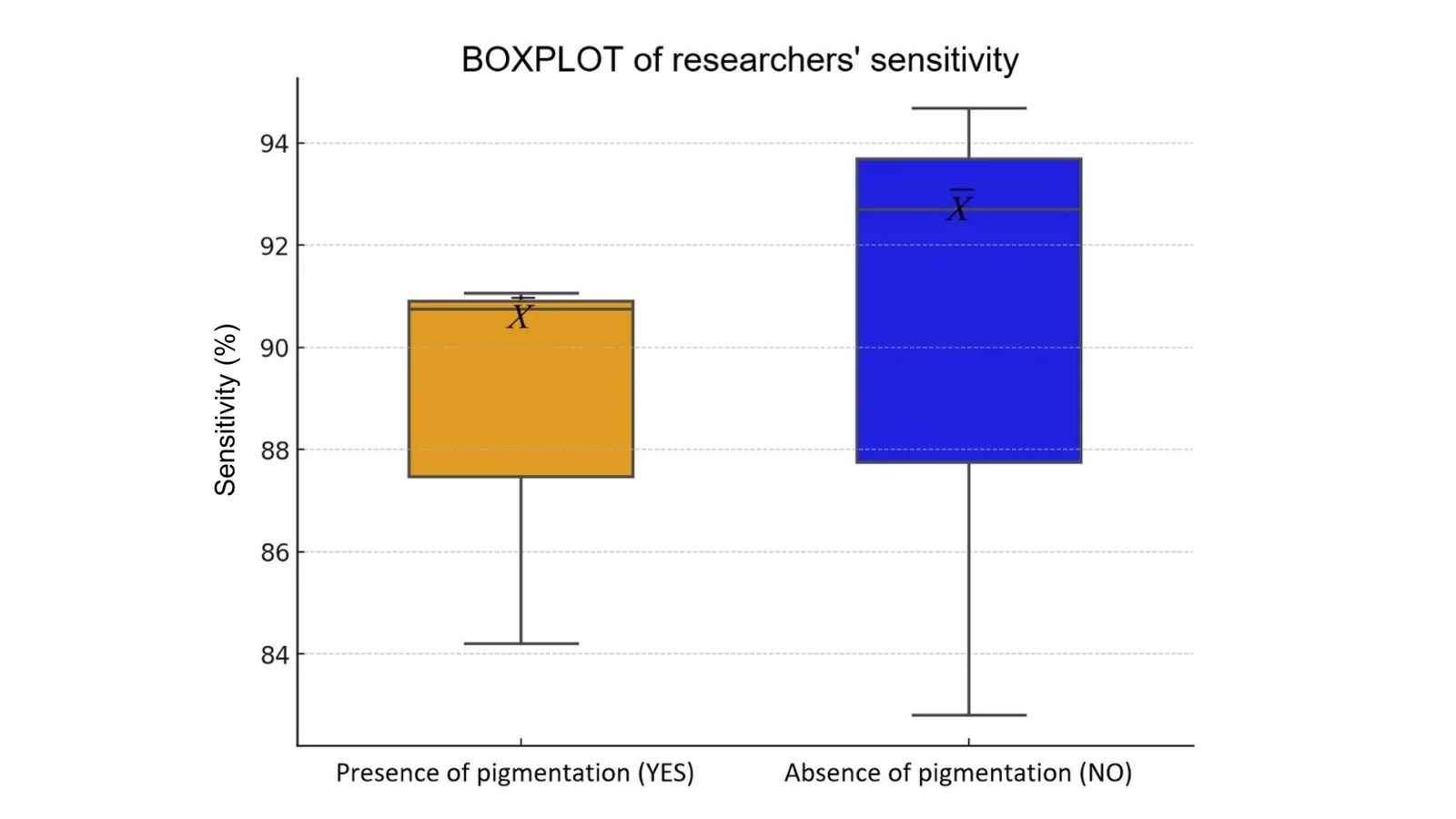
BOXPLOT of researchers’ sensitivity.

### Prediction of post-sclerotherapy hyperpigmentation

The authors observed a strong correlation between Skin Code Score scores and the values provided by the SHI. Patients with a Skin Code Score of 12 points were associated with a 99.8% probability of PSH (>99% accuracy). Patients with a Skin Code Score of 8 had a 76% probability, with 97.5% accuracy. The Skin Code Score and SHI model exhibited strong agreement for forecasting PSH, maintaining predictive validity up to 30 days post-treatment. Notably, patients with a Skin Code Score above eight consistently developed hyperpigmentation, confirming the effectiveness of these predictive methods.

## DISCUSSION

Recent evidence-based guidelines^24^ emphasize that aesthetic complaints and adverse effects related to sclerotherapy, particularly hyperpigmentation, directly influence patient satisfaction and adherence to treatment in chronic venous disease. In this context, development of objective and reproducible clinical tools capable of identifying patients at higher risk for post-sclerotherapy hyperpigmentation may contribute to individualized therapeutic planning and better patient counseling. The findings of the present study align with the current movement toward more standardized and evidence-based approaches in phlebology.^24^

Post-sclerotherapy hyperpigmentation (PSH) is the most frequent adverse effect of sclerotherapy, yet predicting who will develop it remains a challenge.^12^ It is more common in individuals with darker skin tones, leading to emotional distress and reduced confidence. Persistent hyperpigmentation can overshadow the success of sclerotherapy, causing frustration and reducing trust in treatment. To addressing this issue, both the vascular condition and the pigmentation process must be managed to improve patient experience and adherence.^25^

Patients with a Skin Code Score above eight consistently developed hyperpigmentation, confirming the effectiveness of these predictive methods. A score of 12 was associated with a 99.8% probability of PSH, whereas a score of 8 corresponded to a 76% probability, reinforcing the reliability of these tools for clinical decision-making.

The κ range indicated strong interobserver agreement, demonstrating high consistency between evaluators. The p-value shows that most comparisons presented statistically significant differences, indicating that the agreement coefficients are robust and little influenced by chance.

The Skin Code Score is an innovative tool that enhances prediction of hyperpigmentation after sclerotherapy. These practical and straightforward criteria make it a valuable resource for vascular physicians. The score demonstrated strong reliability, with high inter-rater agreement. It also showed results comparable to those of established pigmentation risk calculators, reinforcing its effectiveness for managing treatment expectations and optimizing patient care.

This study demonstrates the feasibility of using the Skin Code Score to predict hyperpigmentation in patients undergoing sclerotherapy for telangiectasias and reticular veins.

### Study strengths, limitations, and future directions

The Skin Code Score is simple to use and reproducible, making it a practical tool for vascular physicians. A key strength was its comparison with dermatologist evaluation, ensuring clinical relevance. Additionally, participating physicians underwent calibration training, achieving over 99% reproducibility and reducing assessment variability.

The Skin Code study has limitations. The sample is small, comprising twenty-one women selected by convenience sampling, since this is a pilot study, limiting generalizability. A total of 114 patients screened for eligibility were excluded because they had a CEAP classification higher than that established for the study, reflecting the chronic venous disease profile of the community evaluated. This exclusion factor was decisive in reducing the number of patients included in the study.

Future research with larger and more diverse groups is needed to validate findings. These efforts should focus on developing standardized protocols for incorporating the Skin Code Score into routine practice. Using the score to guide patient selection and preventive strategies could enhance treatment outcomes and satisfaction.^25^ Ongoing research and technological innovation will ensure its broader adoption in phlebology.

## CONCLUSIONS

The Skin Code Score proved to be a reliable, easy-to-use, and highly reproducible clinical tool for predicting PSH in patients with CVD classified as C1. The score demonstrated high sensitivity and specificity, allowing objective stratification of PSH risk based on clinical parameters available in outpatient practice. The findings demonstrate that the Skin Code exhibited good agreement with the SHI, corroborating its clinical accuracy in PSH cases.

## Data Availability

All data generated or analyzed are included in this article and/or in the supplemental material.
